# Comparison of the Efficiency of Hyperspectral and Pulse Amplitude Modulation Imaging Methods in Pre-Symptomatic Virus Detection in Tobacco Plants

**DOI:** 10.3390/plants12223831

**Published:** 2023-11-12

**Authors:** Alyona Grishina, Oksana Sherstneva, Anna Zhavoronkova, Maria Ageyeva, Tatiana Zdobnova, Maxim Lysov, Anna Brilkina, Vladimir Vodeneev

**Affiliations:** 1 Department of Biophysics, National Research Lobachevsky State University of Nizhny Novgorod, 23 Gagarin Avenue, 603950 Nizhny Novgorod, Russia; 79159532707@yandex.ru (A.G.); ann.zhavoronkova38.95@gmail.com (A.Z.); t.zdobnova@mail.ru (T.Z.); internal_horizon@mail.ru (M.L.); v.vodeneev@mail.ru (V.V.); 2 Department of Biochemistry and Biotechnology, National Research Lobachevsky State University of Nizhny Novgorod, 23 Gagarin Avenue, 603950 Nizhny Novgorod, Russia; ageyevamaria@gmail.com (M.A.); annbril@mail.ru (A.B.)

**Keywords:** *Nicotiana benthamiana*, biotic stress, potato virus X, pre-symptomatic detection, chlorophyll fluorescence imaging, hyperspectral imaging

## Abstract

Early detection of pathogens can significantly reduce yield losses and improve the quality of agricultural products. This study compares the efficiency of hyperspectral (HS) imaging and pulse amplitude modulation (PAM) fluorometry to detect pathogens in plants. Reflectance spectra, normalized indices, and fluorescence parameters were studied in healthy and infected areas of leaves. Potato virus X with GFP fluorescent protein was used to assess the spread of infection throughout the plant. The study found that infection increased the reflectance of leaves in certain wavelength ranges. Analysis of the normalized reflectance indices (NRIs) revealed indices that were sensitive and insensitive to infection. NRI_700/850_ was optimal for virus detection; significant differences were detected on the 4th day after the virus arrived in the leaf. Maximum (F_v_/F_m_) and effective quantum yields of photosystem II (*Φ*_PSII_) and non-photochemical fluorescence quenching (NPQ) were almost unchanged at the early stage of infection. *Φ*_PSII_ and NPQ in the transition state (a short time after actinic light was switched on) showed high sensitivity to infection. The higher sensitivity of PAM compared to HS imaging may be due to the possibility of assessing the physiological changes earlier than changes in leaf structure.

## 1. Introduction

Pathogens are a major problem in agricultural production, causing significant economic losses. Increasing the proportion of agricultural products grown indoors exacerbates the problem, as favorable conditions are created for the proliferation of pathogens. Crop pathogens include various groups of organisms: viruses, bacteria, fungi, oomycetes, and nematodes. Each of them has its own characteristics regarding the method and site of entry into the plant, infection strategy (intracellular or extracellular), and preferred target tissues and organs (xylem, phloem, roots, or leaves). Despite significant differences, all of these pathogens cause significant crop losses by reducing plant health and productivity, as well as increasing post-harvest spoilage [1,2,3].

The importance of the problem of crop diseases has led to significant efforts aimed at solving it. A successful strategy for combating pathogens involves their early detection, since any delay, as well as incorrect identification of the disease, can lead to massive damage to plants. Detection methods include traditional visual assessment of plant diseases by human raters, molecular genetics techniques, and serological and optical remote sensing methods [4,5,6]. The latter are based on assessing the optical properties of a plant in various regions of the electromagnetic radiation spectrum and using sensors that measure reflectance, temperature, or fluorescence. There are a large number of works devoted to the development of remote optical monitoring methods for detecting pathogens. Development and testing of these methods are carried out both in laboratory and field conditions [7,8,9]. The results are summarized in relevant reviews [7,10,11,12,13]. Some studies evaluate the possibility of not only identifying the presence of a pathogen, but also determining the nature of the infection agent, i.e., differentiating diseases. In particular, the method of hyperspectral (HS) imaging has been used to differentiate such pathogens as *Cercosporosa beticola*, *Uromyces betae*, *Erysiphe betae* in beet plants [14] and *Blumeria graminis*, *Puccinia hordei*, *Pyrenophora teres* in barley plants [15].

Despite the significant progress achieved in the development of optical methods for pathogen detection, there are a number of unresolved issues and limitations. In particular, when artificially infecting plants, observations are usually carried out in the inoculated leaf. The observed changes may differ from the development of the disease under natural conditions, since artificial infection is often accompanied by significant mechanical damage to the leaf, and the infectious agent of the disease is injected in a large dose. More relevant is the study of the systemic spread of infection. Despite the fact that the proportion of such works is small, we can talk about the efficiency of such methods as HS imaging [16,17] and registration of chlorophyll fluorescence in pulse-modulated mode (PAM imaging) [18,19,20] in the detection of systemic infection.

Another important issue is that the vast majority of studies evaluate the efficiency of the applied approach in detecting pathogens at the symptomatic stage of the disease, when visual signs of the infection occur. On the other hand, successful localization of disease foci and reduction of the number of agrochemicals applied require the detection of pathogens at a pre-symptomatic stage. Some studies have demonstrated the potential of optical methods in the early detection of pathogens. In particular, HS imaging was successfully used for the detection of TYLC (tomato yellow leaf curl virus) [21] and ToCV (tomato chlorosis virus) [22] in tomato, as well as *Puccina striiformis* in wheat [23]. The fluorescence imaging method was used for pre-symptomatic detection of infection development in ginseng (*Panax quinquefolius* L.) [24], beans (*Phaseolus vulgaris* L.) [25], and melon (*Cucumis melo* L.) [19]. Our previous study also demonstrated the efficiency of PAM imaging in detecting potato virus X (PVX) in tobacco (*Nicotiana benthamiana*) at the pre-symptomatic stage [26].

The results available today indicate that HS and PAM imaging methods can solve the problem of pathogen detection at the pre-symptomatic stage. At the same time, a direct comparison of the efficiency of these optical methods has been carried out only in a few studies. The results of the work [8] showed that the hyperspectral method can distinguish between healthy and infected areas with the appearance of visible symptoms on wheat spikelets, and PAM imaging can distinguish them a day earlier. The potential of multispectral and PAM imaging in detecting the fungus *Botrytis cinerea* was assessed for tomato plants, and changes in PAM parameters were detected earlier compared to reflectance indices [27]. The work of Berdugo et al. [28] showed the high efficiency of both HS and PAM imaging in detecting cucumber mosaic virus (CMV), cucumber green mottle virus (CGMV), and powdery mildew *Sphaerotheca fuliginea* in cucumber plants. However, the studies were conducted at the symptomatic stage of the disease, which limits the use of the results obtained in relation to early detection of diseases.

In this work, we compared the efficiency of HS and PAM imaging for early detection of pathogens using monitoring of the systemic spread of PVX in tobacco plants.

## 2. Results

### 2.1. PVX Spread in Tobacco Plants

**Figure 1:**
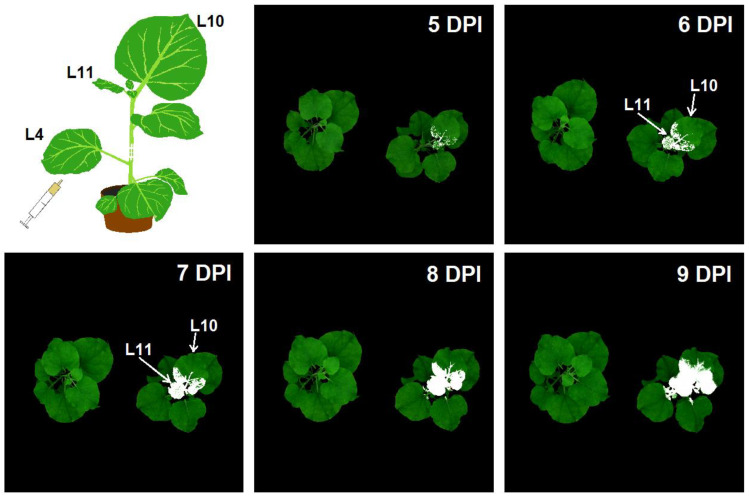
Systemic spread of PVX-GFP throughout a *N. benthamiana* plant on days 5–9 after inoculation (DPI) of the 4th (bottom) leaf. RGB images of whole plants (control plant on the left, infected plant on the right) with overlaid fluorescent images obtained using surface fluorescence imaging are shown. The GFP fluorescence signal is indicated in white. Top left image shows a plant (*N. benthamiana*) diagram with an inoculated leaf (L4) and uninoculated, systemically infected leaves (L10 and L11).

The spread of PVX was detected due to the presence of GFP in its capsid. Images illustrating the systemic spread of the virus through non-inoculated leaves of the plant are shown in Figure 1. Spread into non-inoculated leaves was observed on the 5th day after agroinfiltration of the fourth lower leaf. The virus spread to younger, upper leaves, primarily to the 10th and 11th leaves of tobacco plants. The youngest, small-sized 11th leaf was immediately completely infected. In the larger 10th leaf, the virus was detected at 5th day post-inoculation (DPI) at the base of the midvein, then the virus spread through the leaf veins and moved into the areas between the veins. Symptoms of the disease, which manifested mainly in the formation of a net followed by a partial change in color at later stages of the disease, became visible 4–5 days after the first detection of fluorescence of viral particles (8–9 DPI), when most of the leaf was infected. Taking into account the nature of the development of the infection, which manifested itself in a gradual (over several days) increase in the affected area without pronounced visual symptoms, we chose the 10th leaf as the main object of observation using HS imaging and PAM imaging.

### 2.2. Detection of Viral Infection in N. benthamiana Leaves Using the Hyperspectral Method

**Figure 2:**
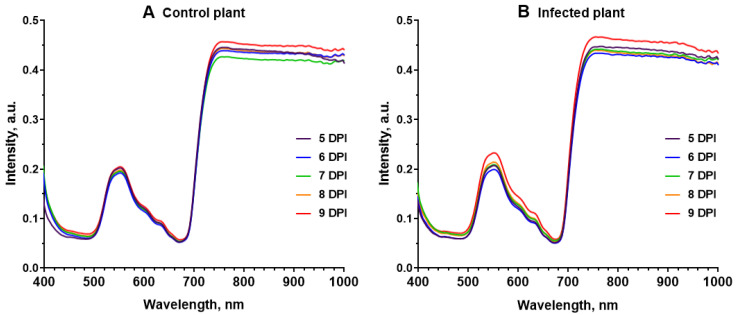
Dynamics of the reflectance spectra of the 10th leaf of the control plant (**A**) and the infected area of the 10th leaf of the inoculated plant (**B**). The curves in the diagrams represent average spectra (*n* = 6).

The reflectance spectra of the 10th leaf of control and infected plants were analyzed to assess the efficiency of the HS imaging method in the early detection of viral infection in *N. benthamiana* plants. Identification of the infected area of the leaf was based on the GFP fluorescent signal of the virus particles. The reflectance spectra of leaves of healthy plants had characteristic low values in the visible range with a maximum in the green region of the spectrum and high values in the near-IR (near-infrared) region with a sharp increase in the red edge (Figure 2A and Figure S1A). The development of infection at the pre-symptomatic stage did not cause dramatic changes; the spectra of infected leaves had the same characteristic features. At the same time, it can be seen that the infected leaf, in contrast to the leaf of the control plant, is characterized by changes in the spectrum with increasing time after infection; the most pronounced changes were observed in the green and red ranges of the spectrum (Figure 2B and Figure S1B).

**Figure 3:**
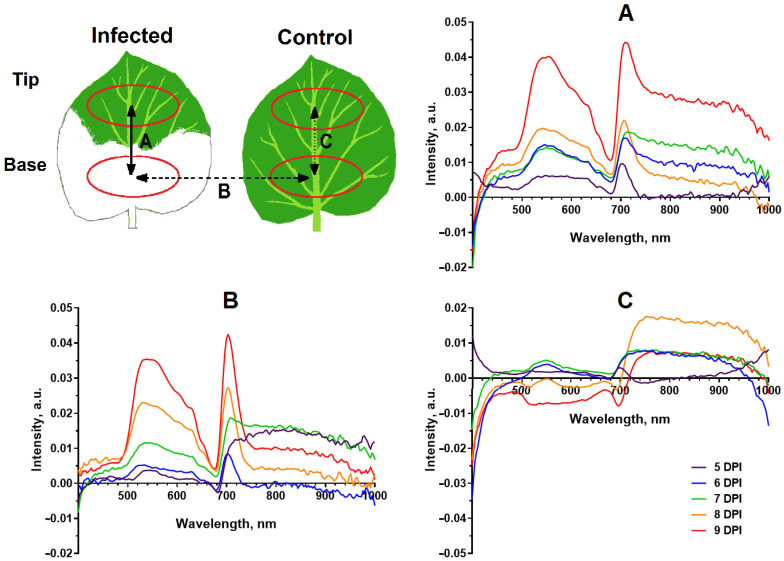
Dynamics of the difference between the spectra of the infected and healthy areas of the 10th leaf of the inoculated plant (**A**) (solid line), the difference between the spectra of the infected area of the inoculated plant and the base of the leaf of the control plant (**B**) (dashed line), the difference between the spectra of the base and the tip of the control plant (**C**) (dotted line). The curves in the diagrams represent the averaged differences between the spectra (*n* = 6). Top left image shows a diagram of the 10th leaf of the *N. benthamiana* plant indicating the regions of interest used in the experiment.

Figure 3 and Figure S2 show the difference in spectra between the infected (basal) and healthy (tip) regions of the 10th leaf of an inoculated plant (A, solid line), the basal parts of a leaf of an inoculated and control plant (B, dashed line), and the basal part of a leaf and the tip of a control plant (C, dotted line). Infection caused an increase in reflectance in the wavelength ranges 510–630 nm and 690–720 nm. The noted differences grew with increasing time after the arrival of the virus in the leaf under study. Along with this, there was a signal attenuation in the range of 400–420 nm. Figure 3C shows that the difference between the base and tip of the control leaf was very small, except in the range of 400–420 nm. This suggests that the difference in the spectra of the infected and healthy areas of the leaf in the ranges of 510–630 nm and 690–720 nm was actually caused by viral infection, and not by differences in reflectance in the older (base) and younger (tip) parts of the same leaf.

**Figure 4:**
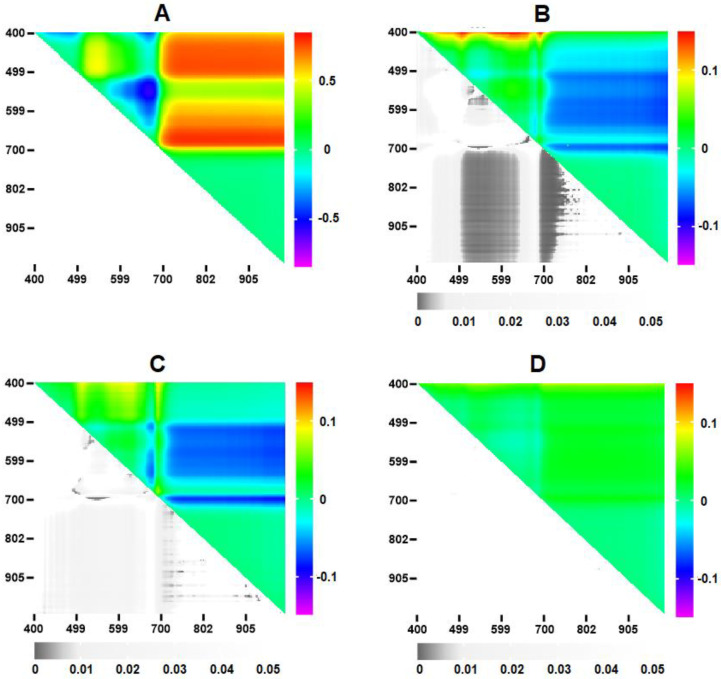
Heat maps of the NRIs of the control leaf (**A**), ΔNRI between the infected and healthy areas of the 10th leaf on the 5th day after the arrival of the virus (9 DPI) (**B**), ΔNRI between the infected area in the 10th leaf of the inoculated plant and the base of the 10th leaf of the control plant (**C**), ΔNRI between the base and the tip of the 10th leaf of the control plant (**D**). The vertical color scale reflects the NRI (**A**) or the ΔNRI value (**B**–**D**); the horizontal gray scale reflects the level of statistical significance of the differences; ΔNRI was compared to zero (*n* = 6).

In addition to reflectance spectra, we also analyzed normalized reflectance indices (NRI = (λ_1_ − λ_2_)/(λ_1_ + λ_2_)), which are reliable tools for analyzing infection-induced changes in reflectance parameters [29,30,31,32]. A heat map of indices for the 10th leaf of the control plant is shown in Figure 4A. The supplementary section (Figure S3) also shows NRI heat maps of healthy and infected areas of the 10th leaf of the inoculated plant. As with the spectra, the indices did not show dramatic changes during the pre-symptomatic stage of infection.

Distinctions in NRIs become visible when constructing heat maps of the differences in indices (ΔNRI) of infected and healthy leaves (Figure 4B–D). Three areas with the maximum differences can be identified on the heat map of ΔNRI between the infected and healthy parts of the leaf: (i) λ_1_ (400–410) nm and λ_2_ (550–700) nm, (ii) λ_1_ (510–630) nm and λ_2_ (750–1000) nm, and (iii) λ_1_ (690–710) nm and λ_2_ (750–1000) nm. The first area was also the maximum for ΔNRI between the basal and tip parts of the leaf of the control plant.

**Figure 5:**
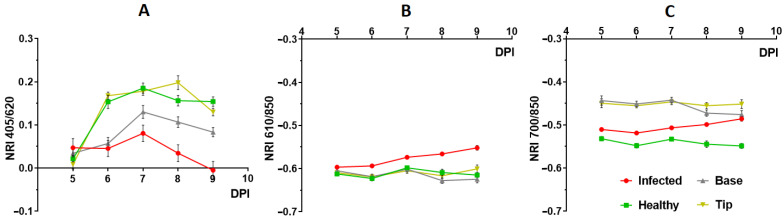
Dynamics of NRIs at 5–9 DPI in different areas of the 10th leaf of the inoculated and healthy plant. NRI_405/620_ (**A**), NRI_610/850_ (**B**), NRI_700/850_ (**C**). Mean values ± SEM are shown (*n* = 6).

We identified those wavelengths for which the differences between infected and healthy parameters were maximum in the spectrum ranges described above. For the first region, these wavelength values were: λ_1_ 405, λ_2_ 620 (NRI_405/620_); for the second region—λ_1_ 610, λ_2_ 850 (NRI_610/850_), for the third—λ_1_ 700, λ_2_ 850 (NRI_700/850_). The dynamics of the NRI values at different DPI is shown in Figure 5A–C. There are significant changes in NRI_405/620_ value at different observation days for both infected and uninfected leaves. The NRI_700/850_ and NRI_610/850_ indices in uninfected leaves remained almost unchanged over the observation period.

**Figure 6:**
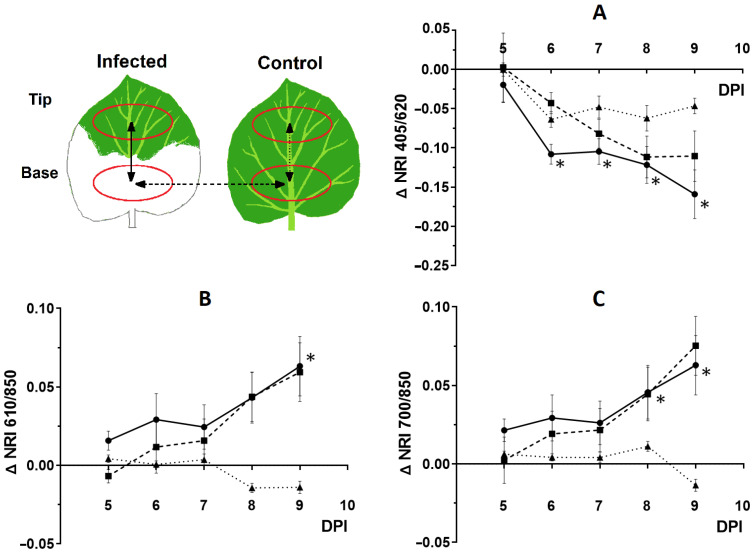
Dynamics of ΔNRIs between different areas of the 10th leaf of the inoculated and control *N. benthamiana* plant at 5–9 DPI. Top left image shows a diagram of the 10th leaf indicating the regions of interest used in the experiment. Shaded arrows, similar to the corresponding lines in the graphs, show regions of interest (ROIs), between which the difference in spectra was calculated. (**A**) ΔNRI_405/620_, (**B**) ΔNRI_610/850_, (**C**) ΔNRI_700/850_. The differences in spectra between the infected (basal) and healthy (tip) regions of the 10th leaf of an inoculated plant (solid line), the basal parts of a leaf of an inoculated and control plant (dashed line), and the basal part of a leaf and the tip of a control plant (dotted line). Mean values ± SEM are given (*n* = 6). * indicates statistically significant differences between values in infected and healthy areas or differences in ratios from 1 (*p* < 0.05).

To identify differences between infected and healthy areas, time courses of ΔNRI were plotted (Figure 6A–C). NRI_405/620_ showed statistically significant differences already at 7 DPI (2nd day of virus arrival in the leaf), while NRI_700/850_ and NRI_610/850_ did so at 8 and 9 DPI, respectively. NRI_700/850_, which has high sensitivity and is independent of age-related changes, was subsequently used to compare the efficiency of the optical methods under study.

### 2.3. Detection of Viral Infection in N. benthamiana Leaves Using the PAM Method

**Figure 7:**
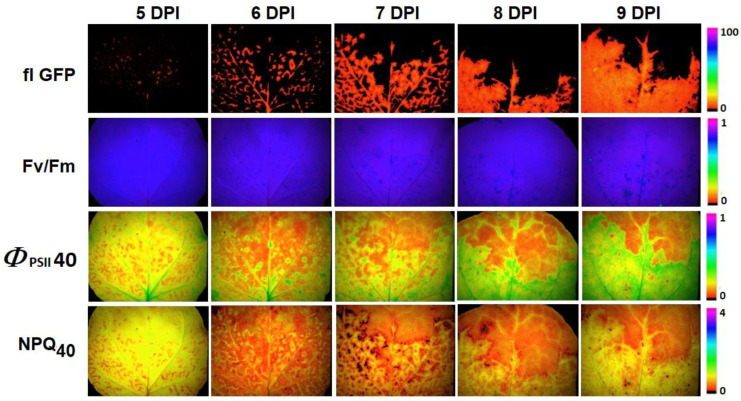
Images of the 10th tobacco leaf, systemically infected with PVX-GFP (PVX containing the GFP protein in its capsid), at different DPI: flGFP—fluorescent images of the spread of viral particles with GFP in the capsid along the leaf (λ_ex_ 460 nm, λ_em_ 500–540 nm); F_v_/F_m_—F_v_/F_m_ images obtained after the first saturation pulse was switched on; *Φ*_PSII40_—*Φ*_PSII_ images obtained 40 s after actinic light was switched on; NPQ_40_—NPQ images obtained 40 s after actinic light was switched on.

The next step was to study the effect of infection on chlorophyll fluorescence using PAM imaging. The parameters measured included maximal quantum efficiency of PSII (F_v_/F_m_), quantum yield of PSII photochemical reactions in the actinic light (*Φ*_PSII_), photochemical quenching (qP), non-photochemical quenching (NPQ, and qN). *Φ*_PSII_, qP, NPQ, and qN were recorded both in the light-adapted state and in the transition state—40 s after the actinic light was switched on. The latter, as shown by our previous study, provides the greatest contrast between infected and healthy areas [26]. Images of F_v_/F_m_, as well as *Φ*_PSII_ and NPQ in the transition state, along with RGB and fluorescent images, are presented in Figure 7. The increase in the area of infection, which was recorded based on the fluorescence of the GFP-labeled virus, was accompanied by an increase in the area of changed chlorophyll fluorescence values.

**Figure 8:**
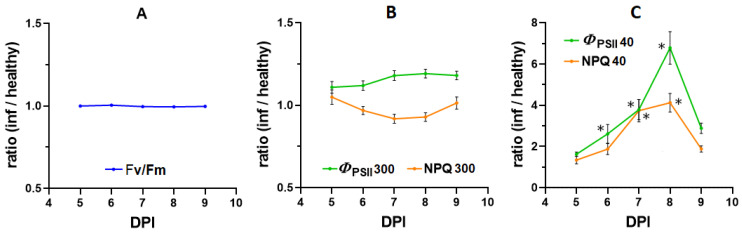
Dynamics of the ratio of the values of chlorophyll fluorescence parameters in the infected area to the healthy area of the 10th leaf of the inoculated tobacco plant. (**A**) F_v_/F_m_ ratios. (**B**) Ratio of photosystem II quantum yield (*Φ*_PSII_) and non-photochemical fluorescence quenching (NPQ) determined at steady state (300 s after actinic light was switched on). (**C**) Ratio of *Φ*_PSII_ and NPQ in the transition state (40 s after actinic light was switched on). Values are means ± SEM (*n* = 6). * indicates statistically significant differences between values in infected and healthy areas or differences in ratios from 1 (*p* < 0.05).

Figure 8 shows the dynamics of chlorophyll fluorescence parameters in the regions of interest on the 10th leaf after its infection depending on time. The initial (zero-time) fluorescence quantum yield (F_o_) and maximal fluorescence quantum yield (F_m_) also did not change throughout the experiment (Figure S4). For steady-state levels of NPQ_300_ and YII_300_ (300 s after switching on the actinic light), the ratios were close to 1 and unchanged throughout the whole experiment, which indicates the low efficiency of using steady-state chlorophyll fluorescence parameters for virus detection. In the transition state (40 s after the actinic light was switched on), *Φ*_PSII40_ and NPQ_40_ showed the greatest differences between the regions of interest. Similar dynamics were also recorded for qP and qN (Figure S5). These areas were significantly different from each other already on the 2nd day of the virus’s arrival into the leaf (6 DPI); the differences grew over time. The differences became much smaller on 5th day (9 DPI), which can be associated with an increase in the area of the leaf affected by the virus and a decrease in the healthy part of the lamina. *Φ*_PSII_, which exhibited the maximum differences, was subsequently used to compare the efficiency of the PAM and hyperspectral methods.

### 2.4. Comparison of the Efficiency of Detection Methods

**Figure 9:**
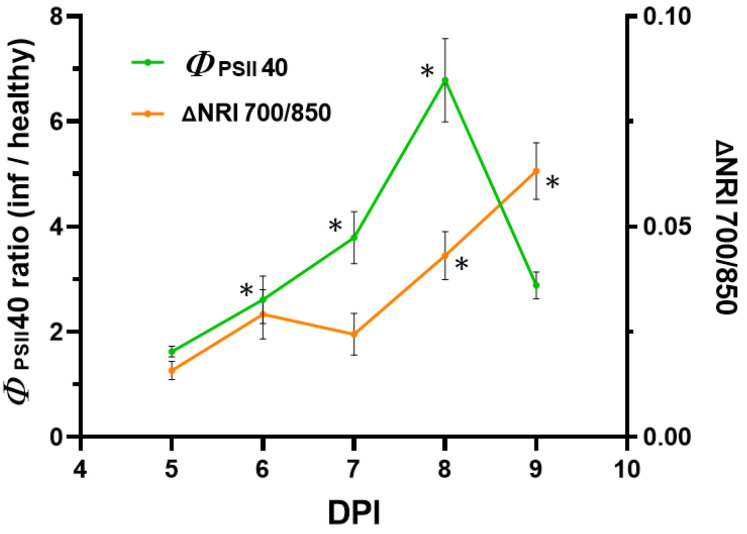
Comparison of *Φ*_PSII40_ and ΔNRI (λ_1_ 700, λ_2_ 850) efficiency for the detection of viral infections in *N. benthamiana* leaves. Values are means ± SEM (*n* = 6). * indicates statistically significant differences between values in infected and healthy areas or differences in ratios from 1 (*p* < 0.05).

The next step in the work was to compare the efficiency of HS and PAM imaging in the pre-symptomatic detection of pathogens. Figure 9 shows the dynamics of the most effective parameters identified as a result of studying each method. A gradual increase was shown for the value of the normalized index determined using HS imaging, reaching a maximum on the 9th day (5th day of virus arrival in the leaf, the last day of measurements). Statistically significant differences between infected and healthy areas of the leaf were observed at 8 DPI (4th day of virus arrival in the leaf). For *Φ*_PSII_ (a parameter determined by PAM imaging), the differences grew faster. A statistically significant difference occurred at 6 DPI (2nd day of virus arrival in the leaf).

## 3. Discussion

The presence of a GFP-labeled virus made it possible to accurately determine the localization and dynamics of PVX spread. The virus spread from the inoculated leaf (leaf 4) to the upper, young leaves (primarily 10th and 11th leaves), while the virus was absent in the lower leaves, which were closer to the inoculated one. This is due to the fact that the main route of systemic spread of the virus is the phloem [33]. Adult leaves are donors of assimilates transported through the phloem, and young growing leaves are acceptors [34]. The infection affects the youngest leaf (leaf 11) completely within a short time; on the contrary, the area of infection increases over several days in the older 10th leaf, usually not reaching 100%. On the one hand, such features may be due to a large influx of phloem into the youngest leaf, and, on the other hand, to the different immune status of leaves of different ages. In particular, the accumulation of metabolites in the leaf grows with increasing age, providing greater resistance to the virus [35]. The gradual development of infection, combined with the sufficiently large area of the 10th leaf, makes it a convenient model, including for distinguishing changes caused directly by pathogens and growth processes.

Changes in the optical parameters of the leaf caused by the virus were studied in this work by HS and PAM methods. The results of hyperspectral studies revealed a change in the reflectance spectrum of tobacco plant leaves at the pre-symptomatic stage of development of infection caused by PVX. The magnitude of the differences between the reflectance spectra of infected and uninfected leaves in the ranges of 510–630 nm and 690–720 nm rises with increasing time after the virus enters the leaf. Pathogen-induced changes in the reflectance spectrum have been previously recorded for a number of plants: tomato [21,22,36], tobacco [37,38,39], wheat [23,40,41], barley [15,42,43], beets [14,44,45], and potatoes [46,47,48]. The most typical changes in reflectance spectra for plants of various species are an increase in reflectance in the wavelength range 500–700 nm and a decrease in reflectance in the NIR range [21,37,41,43,47,49]. The data we obtained differ somewhat from the typical ones, first of all in the absence of a strong decrease in reflectance in the NIR range of the spectrum. The differences can be explained by the absence of pronounced structural changes in the mesophyll, which are associated with a decrease in reflectance in the NIR [50], at the pre-symptomatic stage of infection, which was studied in this work. Earlier changes in the visible, compared to the NIR, as in this work, were identified during the detection of viruses in tobacco plants in the works of other authors [37,38]. It is assumed that [50] the increase in reflectance in the visible range is caused by a decrease in the content of chlorophyll and other pigments. It should be noted that the decrease in chlorophyll content explains well the rise in reflectance at the symptomatic stage of infection, when signs such as a change in leaf color are clearly visible; at the same time, it is not the main reason for the increase in reflectance in the visible range of the spectrum at the pre-symptomatic stage. This is indicated, in particular, by the absence of differences in the F_m_ value reflecting the chlorophyll content in the infected and healthy areas of one leaf (Figure S5). It can be assumed that the changes in the reflectance spectrum recorded in the work are caused by changes in the content of other pigments and/or changes in the structure of pigment –protein complexes.

Analysis of changes in a wide range of the spectrum allowed us to identify the most informative NRIs for detecting viral infection at an early stage. The indices with the maximum differences between infected and healthy areas of the leaf were NRI_405/620_, NRI_610/850_, NRI_700/850_. The first of them has a strong dependence on the age of the leaves (Figure 5A), which, due to low specificity, limits its use for identifying infected areas. Despite the somewhat lower sensitivity of the NRI_700/850_ and NRI_610/850_ indices, their lack of growth-related changes makes them more promising for early detection of the virus.

Another method studied in the work was PAM imaging, which allows us to directly assess the activity of photosynthesis using various parameters. PAM fluorimetry seems to be a fairly sensitive method for detecting infectious agents inside plants [19,24,51]. The high sensitivity of PAM for pathogen detection at the pre-symptomatic stage has been demonstrated in our previous work [26]. The current results demonstrate that F_v_/F_m_, which reflects the structural integrity of PSII, does not change throughout the experimental period (Figure 7 and Figure 8). Also, the absence of changes between infected and healthy areas is typical for stationary parameters *Φ*_PSII 300_ and NPQ_300_. The maximum differences between the infected and healthy parts of the leaf were recorded for *Φ*_PSII40_ and NPQ_40_, i.e., during the transition state (Figure 8). The infected and healthy areas of the 10th leaf were significantly different from each other in terms of *Φ*_PSII 40_ and NPQ_40_ already on the 2nd day of the virus’s arrival in the leaf (6 DPI), several days before the appearance of visible symptoms. Such differences are due to the faster response of *Φ*_PSII_ and NPQ to the switching on of the actinic light. The rate of light-induced changes in *Φ*_PSII_ and NPQ and the time when they reach a stationary level are determined by the rate of reaching equilibrium in the production and consumption of ATP and NADPH [28]. Acceleration of reaching the stationary level of photosynthetic activity parameters may indicate a higher rate of ATP consumption in infected cells, which may be related to the need for additional energy for defense processes [52]. It should be noted that in other studies, infected plant parts showed contradictory reactions to infection. There could be either a simultaneous decrease in both *Φ*_PSII_ and NPQ [53], or a decrease in *Φ*_PSII_ in parallel with an increase in NPQ recorded at steady state [24,51]. These differences may be explained by the fact that in the vast majority of studies, fluorescence parameters were taken already at late stages of leaf infection, as well as directly in inoculated leaves.

Thus, the results of the present work demonstrate that both HS and PAM imaging methods are effective in the early detection of viral infection at early stages of the disease. A direct comparison of the efficiency of the methods (Figure 9) allowed us to detect the higher sensitivity of the PAM imaging method in virus detection. According to the data obtained, the difference in virus detection time was 2 days. Differences in the sensitivity of the methods are mainly related to the fact that PAM fluorimetry allows us to record directly the activity of a physiological process, i.e., photosynthesis activity [54], and HS imaging can record changes in the composition and structure of the plant leaf, which determine its reflectance [55,56]. Alterations in leaf composition and structure, in turn, are a result of changes in the activity of biochemical and physiological processes occurring in the leaf.

## 4. Material and Methods

### 4.1. Plant Material

The work was carried out on 4–5-week-old *Nicotiana benthamiana* plants, which were grown from seeds in a growth chamber with 70% relative humidity and a 16 h photoperiod (with cool-white lamp, OSRAM, Munich, Germany; 60 μmol m^−2^ s^−1^) at 25 °C.

### 4.2. Infection of Plants by PVX

To infect plants, we used PVX containing the GFP protein in its capsid, which makes it possible to detect the pre-symptomatic spread of viral particles using surface imaging methods [57]. We infected *N. benthamiana* plants with PVX using agrobacterial infiltration with two transformants of *A. tumefaciens* (strain C58C1) with pBin-PVX-GFP (expression of PVX with GFP protein in the plant) and pLH-P19 (suppression of RNA interference) vectors. The Agrobacterium cultures carrying corresponding binary vectors were kindly provided by Prof. A.G. Solovyev (Lomonosov Moscow State University, A.N. Belozersky Research Institute of Physico-Chemical Biology). Agroinfiltration was carried out into the 4th adult tobacco leaf (Figure 1). The detailed procedure for agrobacterial infiltration of plants is described in our previous work [26].

### 4.3. Study of Systemic Spread of PVX in Tobacco Plants

Visible symptoms of a viral infection (mosaic and yellow spots on lamina, leaf curling) were identified by external examination of plants and obtaining RGB images using a Canon EOS 4000D EF-S 18–55mm SLR camera (Canon, Tokyo, Japan).

Pre-symptomatic systemic spread of the virus throughout the plant was monitored by recording the GFP fluorescence signal of virus particles in non-inoculated leaves using PlantExplorerPro+ (PhenoVation, Wageningen, The Netherlands). Whole tobacco plants were placed in a closed chamber, and RGB and fluorescent images were obtained. GFP fluorescence was excited at λ_ex_ 452/45 and detected in the range of λ_em_ 535/43 nm. GFP fluorescence was also recorded using the IMAGING-PAM MINI Version system (Heinz Walz GmbH, Uelzen, Germany) in the case of PAM imaging (λ_ex_ 460 nm, λ_em_ 500–540 nm).

### 4.4. Optical Methods for Early Detection of Viral Infection in Leaves

Monitoring of spectral and fluorescent changes in *N. benthamiana* leaves was carried out in the 10th non-inoculated leaf from 5 to 9 days after agroinfiltration using a hyperspectral camera and a PAM fluorometer.

#### 4.4.1. Hyperspectral Imaging

Hyperspectral imaging of plants was carried out using a Specim IQ hyperspectral camera (Specim, Spectral Imaging Ltd., Oulu, Finland, spectral range 400–1000 nm, 204 spectral bands, sampling interval 3 nm, 0.2 megapixels matrix).

Images of infected and control plants located nearby were obtained simultaneously. The distance between the 10th leaf being examined and the camera was 70 cm. A white reference panel for the Specim IQ hyperspectral camera was used as a reflectance standard to calibrate each measurement. The measurements were carried out in a dark room to avoid the influence of external lighting. Three 150 W halogen lamps were used for lighting, the illumination intensity was 246 µmol m^−2^ s^−1^. The emission spectrum of the lamp is shown in Figure S6.

Five regions of interest (ROIs) of equal area were placed in the infected (leaf base) region of the 10th leaf of the inoculated plant and the same number in the healthy (tip) region; the ROI values within each region were then averaged. Identification of the infected area was based on the GFP signal of the virus in fluorescent images. ROIs were placed on the 10th leaf of the control plant in similar areas (leaf base and tip). Reflectance spectra as well as normalized reflectance indices (NRIs) were used in the analysis. Reflection intensity and index values in each ROI were averaged and used in further analysis. The NRIs were calculated as follows:

(1) $$\mathrm{NRI}=\frac{\lambda_{1\;}-\lambda_{2}}{\lambda_{1}+\lambda_{2}}$$

where λ_1,2_ are the wavelengths in the entire range of the recording spectrum.

The values of the entire set of NRIs in the range of 400–1000 nm were presented as heat maps.

#### 4.4.2. PAM Fluorometry

PAM imaging of the 10th leaf of tobacco plants was carried out using IMAGING-PAM MINI Version (Heinz Walz GmbH, Effeltrich, Germany). The following parameters were measured. Actual quantum yield of PSII photochemical reactions in the actinic light (*Φ*_PSII_), photochemical quenching (qP), non-photochemical quenching (NPQ, qN), maximal quantum efficiency of PSII (F_v_/F_m_), maximal fluorescence quantum yield (F_m_), and initial (zero-time) fluorescence quantum yield (F_0_) [54], calculated using the Equations:

(2) $$\Phi_{PSII}=\frac{F_{m}’-F}{F_{m}’}$$

(3) $$NPQ=\frac{F_{m}-F_{m}’}{F_{m}’}$$

(4) $$qP=\frac{F_{m}’-F}{F_{m}^{’}-F_{0}}$$

and

(5) $$qN=\frac{F_{m}-F_{m}’}{F_{m}-F_{0}}$$

where F_m_ is the maximum fluorescence yield of photosystem II, F_0_ is initial (zero-time) fluorescence in light-adapted samples, and F and F_m_′ are the current and the maximum fluorescence yields of photosystem II under lighting.

To spatially map the effective quantum yield of PSII electron flow, pulses of saturating light were applied every 10 s. The plant was dark-adapted for 15 min before measurements. The detailed procedure and recording protocol are described in [26]. The actinic light intensity was 111 μmol m^−2^ s^−1^.

To analyze PAM images, ROIs were placed in the same way as for hyperspectral imaging (p. 4.4.1).

### 4.5. Statistics

Statistical processing of the results was carried out using MS Excel 2021(Microsoft Corporation, Redmond, WA, USA) and GraphPad Prism software 8.01 (GraphPad Software Inc., San Diego, CA, USA). The average values of the parameters and their standard errors were calculated. The significance of the differences was assessed using the *t*-test. A one-sample t-test was used to determine whether the ratios of parameters in infected and healthy areas and their differences were significantly different from 1 and from 0, respectively. The p-value was considered significant when *p* < 0.05. Eight experimental plants and six control plants were used for the experiment. Measurements were performed on six control and six infected plants.

## 5. Conclusions

The performed study showed that PAM and HS imaging methods make it possible to non-invasively detect viral infection at an early stage of the disease. PAM fluorometry is characterized by greater sensitivity of transient parameters of chlorophyll fluorescence (short time after actinic light is turned on) in comparison with stationary (light-adapted state) parameters. The capabilities of hyperspectral imaging in pathogen detection are based on differences in the reflectance of infected and healthy leaves in certain spectral ranges. The discovered ability of the PAM imaging method to detect a viral infection somewhat earlier in comparison with HS imaging (higher sensitivity of PAM) is probably due to the ability of the PAM method to directly assess the activity of photosynthesis. In addition, changes in the activity of physiological processes caused by stressors precede changes in the composition and structure of the plant.

Having high sensitivity, the PAM imaging method has a number of limitations for its use in large-scale studies. First of all, it is necessary to note the need for a high homogeneity of illumination conditions and a long duration of analysis due, in particular, to the need for dark adaptation. In this regard, the most promising field for this method is breeding programs in which the susceptibility of plant lines to pathogens is assessed (when studies are performed on a limited number of plants). HS imaging has no such limitations and is suitable for large-scale screening studies to identify disease foci in the field. The most informative bands in the reflectance spectrum identified by HS are convenient input data for the development of cheaper multispectral imaging systems.

In general, this work highlights the need for further study of spatial and temporal differences in the response to pathogens at the organ and tissue levels, which will make it possible to decipher the mechanisms of interaction between the pathogen and the host plant and, based on the knowledge obtained, to develop effective strategies for combating crop diseases.

## Data Availability

Data are contained within the article and supplementary materials.

## Supplementary Materials

The following supporting information can be downloaded at: https://www.mdpi.com/article/10.3390/plants12223831/s1. Figure S1: Dynamics of the reflectance spectra of the 10^th^ leaf of the control plant (A) and the 10th leaf of the inoculated plant (B). Mean values ± SEM are given (*n* = 6); Figure S2: Dynamics of the difference between the spectra of the infected and healthy areas of the 10th leaf of the inoculated plant (A), the difference between the spectra of the infected area of the inoculated plant and the base of the leaf of the control plant (B), the difference between the spectra of the base and the tip of the control plant (C). Mean values ± SEM are given (*n* = 6); Figure S3: Heat maps of the NRIs of the infected area (A) and healthy area (B) of the 10th leaf on the 5th day after the arrival of the virus (9 DPI) (*n* = 6); Figure S4: Images of the 10th tobacco leaf, systemically infected with PVX-GFP, at different DPI: flGFP—fluorescent images of the spread of viral particles with GFP in the capsid along the leaf (λ_ex_ 460 nm, λ_em_ 500–540 nm); F_0_–F_0_ images obtained after dark adaptation; F_m_–F_m_ images obtained after dark adaptation; Figure S5: Ratio of *Φ*_PSII_, NPQ, qP, and qN in the transition state (40 s after actinic light was switched on). Values are means ± SEM (*n* = 6); Figure S6: The emission spectrum of the halogen lamps used for HS imaging.
