# Supplementary material for: Comparison of the Efficiency of Hyperspectral and Pulse Amplitude Modulation Imaging Methods in Pre-Symptomatic Virus Detection in Tobacco Plants
Source: Plants (Basel). 2023 Nov 12;12(22):3831. doi: 10.3390/plants12223831 (PMC10674761; doi:10.3390/plants12223831)
Supplement: Supplementary file 1 [file plants-12-03831-s001.zip › plants-2680239-supplementary.pdf]

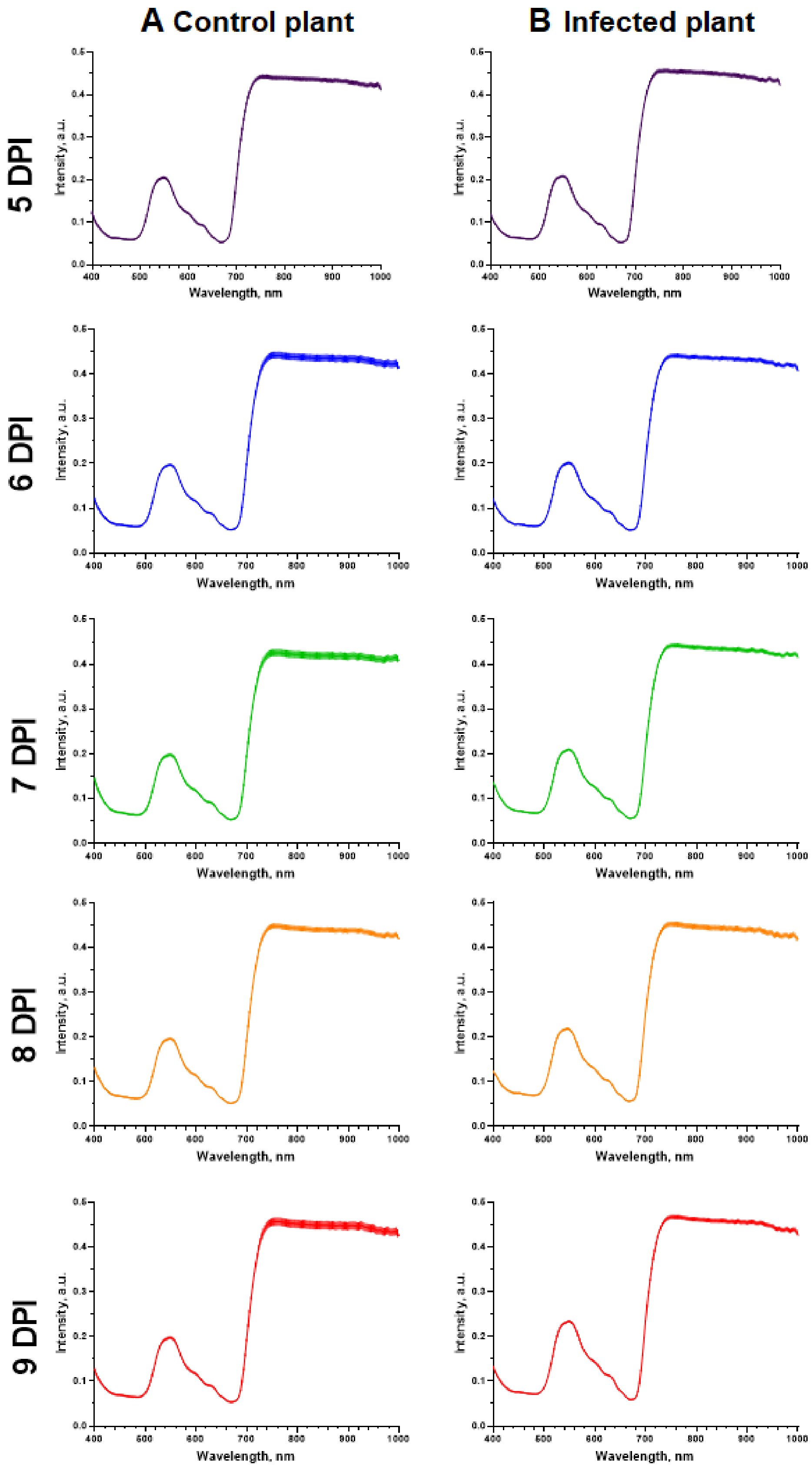

Figure S1. Dynamics of the reflectance spectra of the 10th leaf of the control plant (A) and the 10th leaf of the inoculated plant (B). Mean values ± SEM are given *(n=6)*.

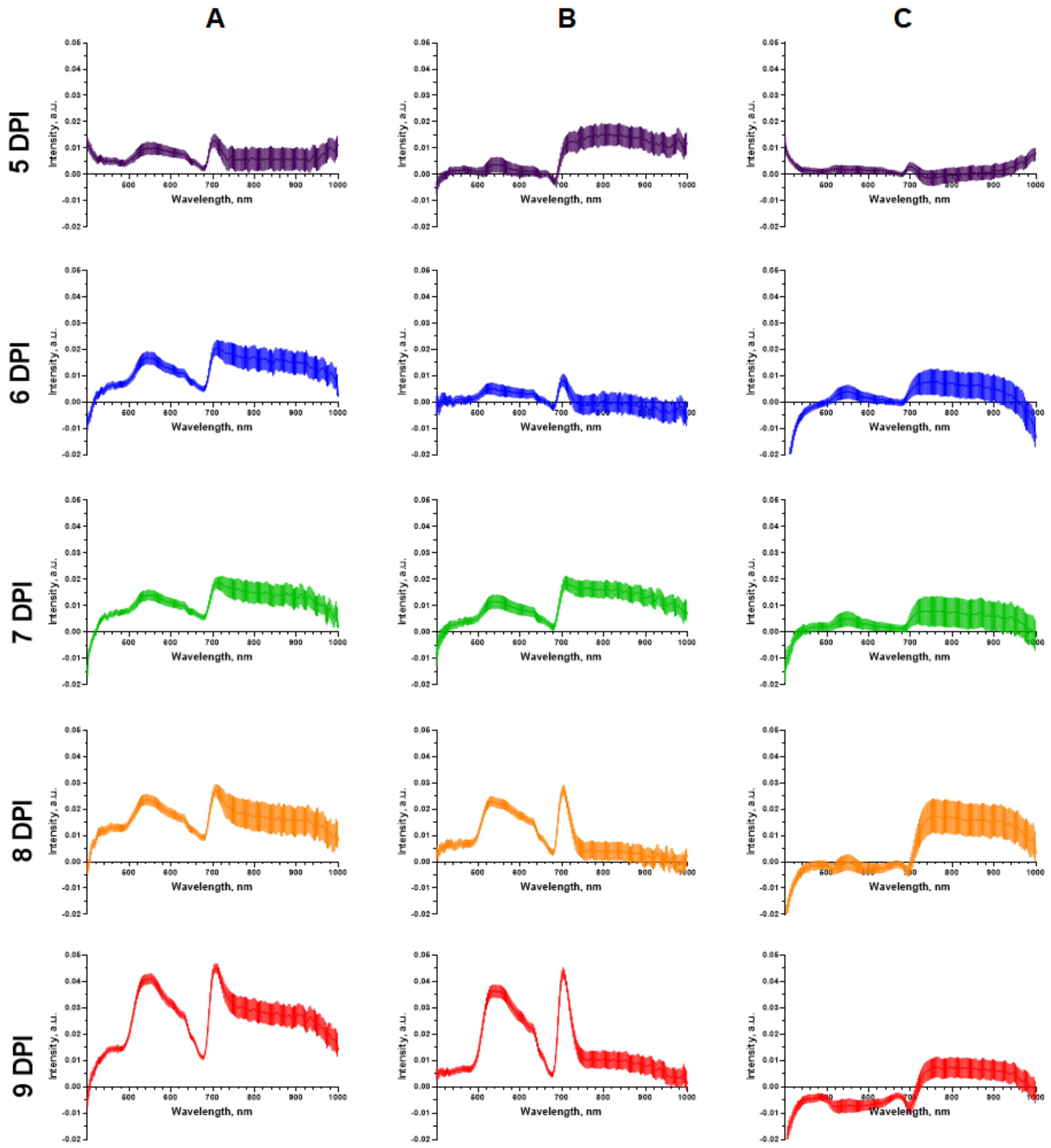

Figure S2. Dynamics of the difference between the spectra of the infected and healthy areas of the 10th leaf of the inoculated plant (A), the difference between the spectra of the infected area of the inoculated plant and the base of the leaf of the control plant (B), the difference between the spectra of the base and the tip of the control plant (C). Mean values ± SEM are given *(n=6)*.

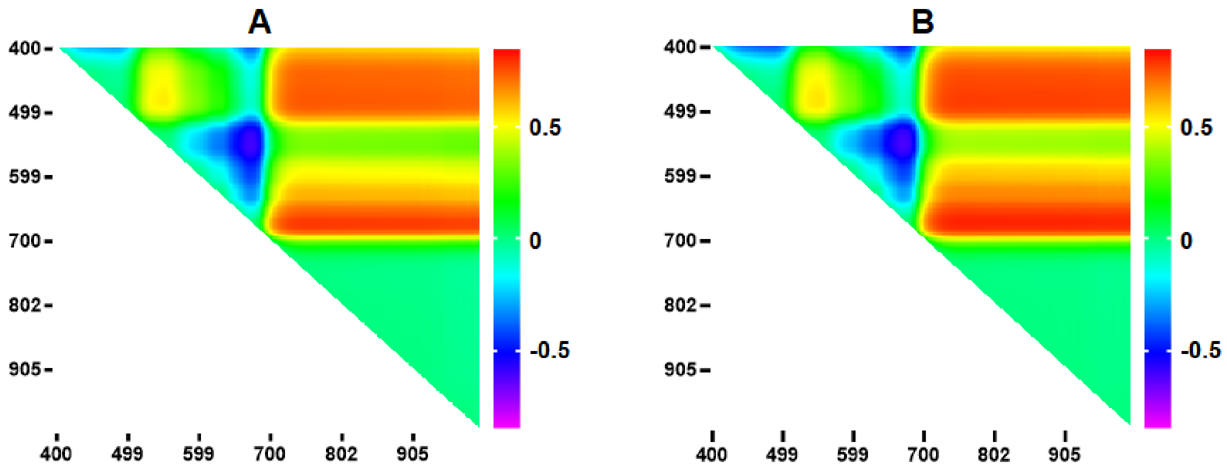

Figure S3. Heat maps of the NRIs of the infected area (A) and healthy area (B) of the 10th leaf on the 5th day after the arrival of the virus (9 DPI) *(n=6)*.

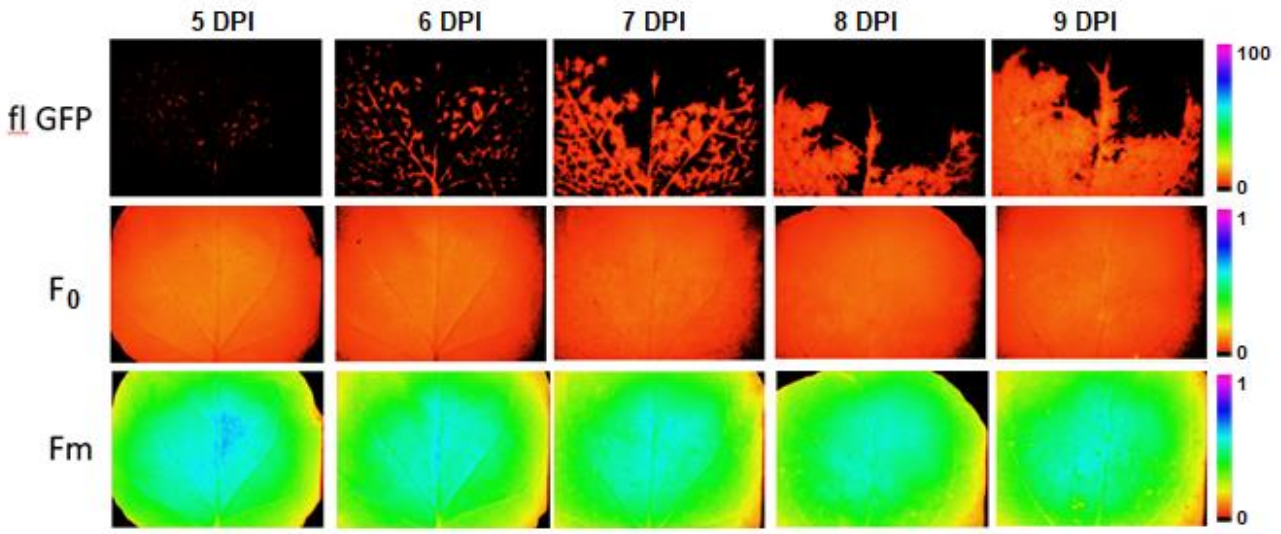

Figure S4. Images of the 10th tobacco leaf, systemically infected with PVX-GFP, at different DPIs: flGFP – fluorescent images of the spread of viral particles with GFP in the capsid along the leaf (λ_{ex} 460 nm, λ_{em} 500–540 nm); F_0 - F_0 images obtained after dark adaptation; F_m – F_m images obtained after dark adaptation.

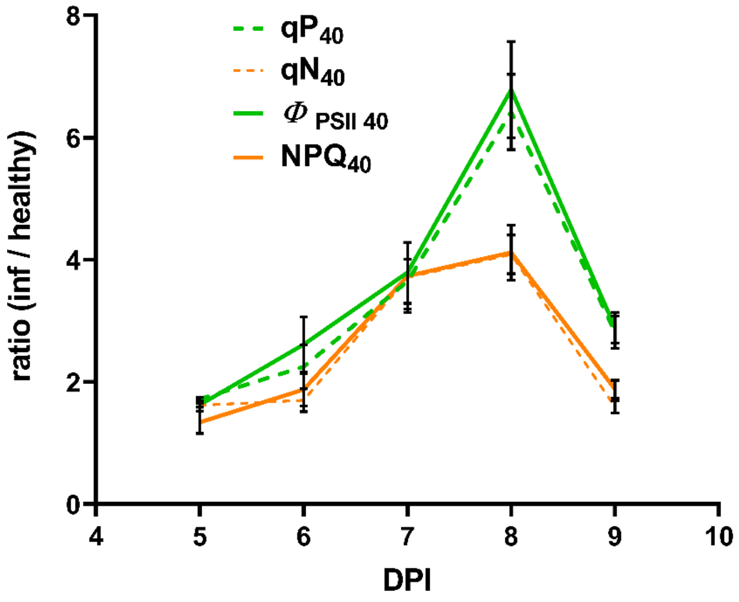

Figure S5. Ratio of Φ_{PSII}, NPQ, qP and qN in the transition state (40 s after actinic light was switched on). Values are means ± SEM *(n=6)*.

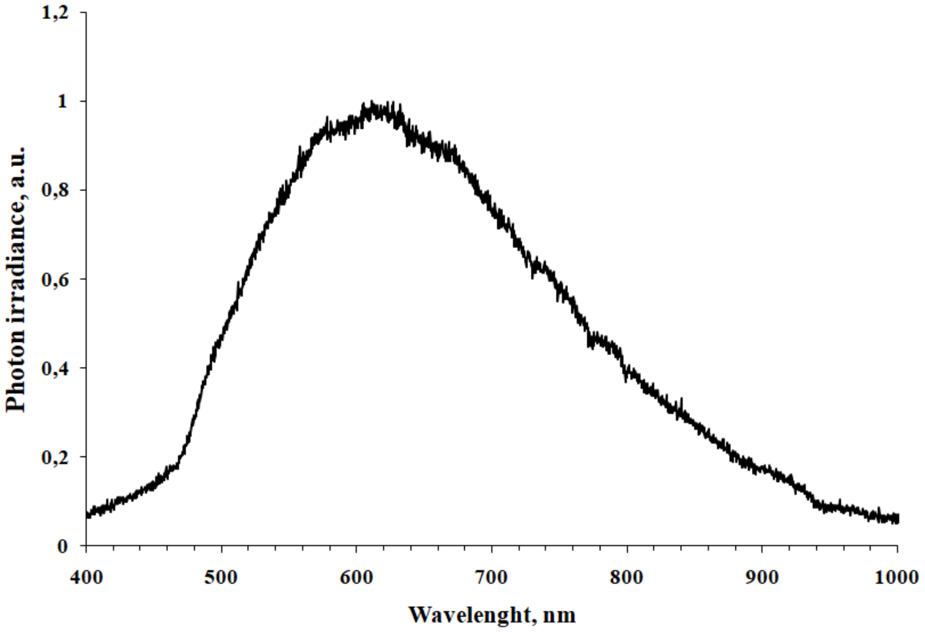

Figure S6. The emission spectrum of the halogen lamps used for HS imaging
